# Metabolic syndrome is independently associated with increased 20-year mortality in patients with stable coronary artery disease

**DOI:** 10.1186/s12933-016-0466-6

**Published:** 2016-10-28

**Authors:** Arwa Younis, Anan Younis, Boaz Tzur, Yael Peled, Nir Shlomo, Ilan Goldenberg, Enrique Z. Fisman, Alexander Tenenbaum, Robert Klempfner

**Affiliations:** 1 The Leviev Heart Center, Sheba Medical Center, Tel Hashomer, Sheba Road 2, 52620 Ramat Gan, Israel; 2 Sakler School of Medicine, Tel Aviv University, Ramat Gan, Israel; 3 Cardiovascular Diabetology Research Foundation, Holon, Israel; 4 Heart Research Follow-up Program, University of Rochester, Rochester, NY USA

**Keywords:** Metabolic syndrome, Stable coronary artery disease, Prognosis, All-cause mortality, Long term outcomes

## Abstract

**Background:**

Data regarding long-term association of metabolic syndrome (MetS) with adverse outcomes are conflicting. We aim to determine the independent association of MetS (based on its different definitions) with 20 year all-cause mortality among patients with stable coronary artery disease (CAD).

**Methods:**

Our study comprised 15,524 patients who were enrolled in the Bezafibrate Infarction Prevention registry between February 1, 1990, and October 31, 1992, and subsequently followed-up for the long-term mortality through December 31, 2014. MetS was defined according to two definitions: The International Diabetes Federation (IDF); and the National Cholesterol Education Program–Third Adult Treatment Panel (NCEP).

**Results:**

According to the IDF criteria 2122 (14%) patients had MetS, whereas according to the NCEP definition 7446 (48%) patients had MetS. Kaplan–Meier survival analysis showed that all-cause mortality was significantly higher among patients with MetS defined by both the IDF (67 vs. 61%; log rank-p < 0.001) as well as NCEP (67 vs. 54%; log rank-p < 0.001) criteria. Multivariate adjusted mortality risk was 17% greater [Hazard Ratio (HR) 1.17; 95% Confidence Interval (CI) 1.07–1.28] in patients with MetS according to IDF and 21% (HR 1.21; 95% CI 1.13–1.29) using the NCEP definition. Subgroup analysis demonstrated that long-term increased mortality risk associated with MetS was consistent among most clinical subgroups excepted patients with renal failure (p value for interaction < 0.05).

**Conclusions:**

Metabolic syndrome is independently associated with an increased 20-year all-cause mortality risk among patients with stable CAD. This association was consistent when either the IDF or NCEP definitions were used.

*Trial registration* retrospective registered

**Electronic supplementary material:**

The online version of this article (doi:10.1186/s12933-016-0466-6) contains supplementary material, which is available to authorized users.

## Background

The metabolic syndrome (MetS) is a constellation of cardiovascular risk factors centered around obesity, abnormal glucose metabolism, hypertension and atherogenic dyslipidemia [1, 2].

These risk factors tend to cluster together in patients, and when they do, they substantially increase the risk for the development of cardiovascular disease [2]. The prevalence of the MetS is increasing, coincident with increasing levels of obesity related to sedentary lifestyles and poor nutrition habits [3–6].

The association of the MetS with increased risk of adverse cardiovascular outcomes, morbidity and mortality is well established [7–13]. However, controversy remains regarding independent character of this association as well as regarding the additional value of the MetS in the risk estimation on top of its individual components. Furthermore, recent studies showed that MetS is associated with an increased risk of cardiovascular mortality and re-infarction in patients with cardiovascular disease [14, 15], however, these studies are mostly limited to patients after a recent acute coronary syndrome (ACS) or after revascularization, and there is limited data regarding patients with stable coronary disease without revascularization procedures [16]. Furthermore, to date the follow-up period in the majority of studies exploring the association of MetS and mortality is less than 3 years [16–21] and these studies have predominantly explored cardiovascular mortality and not all-cause mortality as their primary outcome [16–21].

Thus, limited data exist regarding the association between the presence of MetS and long-term all-cause mortality among patients with stable coronary artery disease (CAD), especially among those who have not undergone prior coronary revascularization or recent ACS. It is unclear whether this association is independent following adjustment for other comorbidities and clinical characteristics. Furthermore, the principle definitions of MetS have not been compared in large cohorts of patients with stable CAD.

Accordingly, the aims of the present study were to: (1) determine the independent association of MetS as defined by the National Cholesterol Education Program (NCEP) vs. the International Diabetes Federation (IDF) criteria with 20-year all-cause mortality outcome; (2) evaluate the heterogeneity of the association between MetS and mortality in important subgroups of patients.

## Methods

### Study population

The present study population comprised patients who were screened for participation in the Bezafibrate Infarction Prevention (BIP) trial between February 1990 and October 1992 and enrolled in the BIP Registry. The design and rationale of the BIP Registry and study were published previously [22, 23]. Of the 15,524 screened patients, only 3090 (20%) proceeded to be randomized in the prospective interventional 6-year BIP study that compared Bezafibrate to placebo. As the intervention period ended more than 15 years ago we decided to include these patients in our analysis cohort.

Briefly, BIP Registry included 15,524 patients aged 40–74 years with stable CAD fulfilling the following inclusion criteria: (1) documented myocardial infarction (MI) in the previous 5 years, (2) symptomatic stable angina pectoris and either a positive myocardial ischemia by radio-nuclear-scintigraphy, or ≥60% stenosis of 1 of the major coronary arteries, demonstrated by coronary angiography, or (3) documented percutaneous transluminal coronary angioplasty (PTCA) or coronary artery bypass grafting (CABG) operation in the preceding 6 months. Exclusion criteria included: diabetes mellitus requiring the use of insulin, severe heart failure, unstable angina, hepatic or renal failure, and current use of lipid-modifying drugs.

All medical examination and biochemical blood-tests, historical medical data, as well as data on drug therapy were prospectively recorded and all vital signs measured.

After exclusion of those patients with missing laboratory values the final data set for the current study comprised of 15,413 patients. Median follow up duration was 20 ± 5 years.

The study was approved by the institute’s internal review board and was performed according to the principles expressed in the Declaration of Helsinki and the ethics policy of the institute.

### Metabolic syndrome definitions

Currently, there are two major definitions for MetS: the International Diabetes Federation (IDF) [24, 25] and National Cholesterol Education Program–Third Adult Treatment Panel (NCEP) [26]. Accordingly, study patients were categorized by the presence or lack of MetS by the two separate definitions.

#### Diagnosis criteria

Patients who presented with three or more of the following five risk factors were defined as having MetS according to the NCEP:

1. Central obesity defined as waist circumference greater than established ethnicity specific values. Since the data regarding waist circumference were not available, for purposes of this analysis we used the accepted body mass index (BMI) above 30 as a criterion for classifying patients as obese [25, 27].
2. Low high-density lipoprotein (HDL) <50 mg/dL among women, and <40 mg/dL among men.
3. Elevated fasting plasma triglycerides (TG) ≥150 mg/dL, or specific treatment for this lipid abnormality.
4. Elevated systolic blood pressure ≥130 mm Hg, or diastolic value ≥85 mm Hg, or treatment of previously diagnosed hypertension.
5. Elevated fasting plasma glucose (FPG) ≥100 mg/dL or previously diagnosed diabetes mellitus.

The definition of the MetS according to the IDF has some modifications as it requires central obesity as an obligatory criterion, and two or more of the other criteria as detailed above. Central obesity can be substituted by BMI >30 [25, 27, 28].

Patients were defined as diabetics based on their medical record diagnosis as prospectively coded at study entry. The same method was applied to the definitions of hypertension, smoking status and other elements of medical history. Patients with diabetes were considered in this analysis as having impaired fasting glucose even if their point measurement of FPG was below 100 mg/dL.

#### Laboratory methods

Blood samples were drawn after at least 12 h of fasting. Cooled samples, collected in the 18 participating centers using standard equipment and procedures, were transferred to the study’s central laboratory. All analyses were performed on a Boehringer Hitachi 704 random access analyzer using Boehringer diagnostic kits. Detailed data on laboratory methods were given in a previous report [29].

### Primary end point

The primary end point of this study was all-cause mortality. Mortality data was obtained by matching the patient’s identification numbers with their vital status available in the National Population Registry of Israel. Each match record was checked for correct identification by matching the date of birth coded during survey enrollment with the date of birth available from the national registry. Patients with missing values or inconsistent matching were excluded from the present analysis (n = 111).

### Statistical analysis

Continuous variables are expressed as mean ± standard deviation (SD), and categorical data are summarized as percentages. The clinical characteristics of the patients at baseline by presence of the MetS were compared with the use of the unpaired t test for continuous variables or Mann–Whitney as appropriate, and the Chi square test for categorical variables.

The Kaplan–Meier method was used to calculate cumulative survival curves for patients with and without MetS and the curves were compared using a Log rank test.

Multivariate Cox proportional hazard regression modeling was used to assess the independent effect of the MetS on the primary end point of all-cause mortality. The following covariates were introduced using the best subset method, following a univariate analysis of all relevant variables: age, gender, smoking status, creatinine concentration, diagnosis of diabetes mellitus (DM), hypertension, heart failure NYHA >2, previous MI or past cerebrovascular accident (CVA). We additionally performed multivariate analysis as described above and included medication (anti-platelets, nitrates, calcium channel blockers, beta blockers and diuretics) as additional covariates.

Proportionality of hazard assumption was verified using Schoenfeld residuals and the log minus log (LML) method. We additionally performed a sensitivity analysis excluding patients randomized to the BIP randomized study (n = 3090).

In order to further explore the independent risk associated with the presence of MetS in pre-specified patient subgroups we performed interaction term analysis by the introduction of an MetS-by-risk-subgroup interaction-term to the multivariate age adjusted Cox model (MetS by age interaction was not further adjusted). The following pre-specified subgroups were explored: age ≥65 years, gender, prior MI, renal dysfunction [serum creatinine >1.5 mg/dL], and New York Heart Association (NYHA) class >2. Interaction analysis is graphically presented in the form of a Forest plot.

Furthermore, in order to confirm our findings, an additional sensitivity analysis was performed, in which the waist circumference\BMI criteria were excluded, and the diagnosis of MetS was made if patients had two out of the four remaining criteria.

Statistical significance was declared for a two-sided p < 0.05. The statistical analysis was performed with IBM SPSS version 20.0 (Chicago, IL, USA) and SAS version 9.2 (SAS institute Inc.) statistical software.

## Results

Based on the criteria of the IDF 2122 (14%) patients had MetS, compared to 13,291 (86%) patients without the MetS, whereas based on the NCEP criteria 7446 (48%) patients had MetS, and 7967 (52%) were considered without. Baseline characteristics of patients with and without MetS according to both definitions are summarized in Table 1.

**Table 1:** Baseline characteristics of the study population by the two metabolic syndrome definitions

	Metabolic IDF definition		*P* value	Metabolic NCEP definition		*P* value
	No (n = 13,291)	Yes (n = 2122)		No (n = 7967)	Yes (n = 7446)	
Age (years)^a^	60 ± 7	59 ± 7	<0.001	60 ± 7	60 ± 7	0.29
Male gender	10,910 (82%)	1540 (73%)	<0.001	6600 (83%)	5850 (78%)	<0.001
Active smoker	1485 (11%)	276 (13%)	<0.001	832 (47%)	929 (53%)	<0.001
HTN	4197 (32%)	948 (45%)	<0.001	2197 (27%)	2948 (40%)	<0.001
DM	2355 (18%)	604 (29%)	<0.001	784 (10%)	2175 (30%)	<0.001
COPD	382 (3%)	70 (3%)	0.27	238 (3%)	214 (3%)	0.85
Past MI	9611 (72%)	1519 (72%)	0.91	5754 (72%)	5376 (72%)	0.92
Past CVA	236 (1.8%)	33 (1.6%)	0.47	131 (1.6%)	138 (1.9%)	0.26
NYHA class >2	753 (5%)	185 (9%)	<0.001	409 (5%)	491 (6.8%)	<0.001
Laboratory values (mg/dL)						
Creat^a^	1.15 ± 0.2	1.11 ± 0.2	<0.001	1.15 ± 0.2	1.14 ± 0.2	0.02
CHO^a^	224 ± 39	228 ± 42	<0.001	212 ± 37	228 ± 41	<0.001
LDL^a^	155 ± 34	155 ± 37	0.73	154 ± 33	156 ± 36	<0.001
Medication						
Bezafibrate	1369 (10%)	193 (9%)	0.10	784 (10%)	778 (10%)	0.22
Placebo	1347 (10%)	210 (10%)	0.68	760 (9.5%)	797 (11%)	0.02
Beta-blockers	4527 (34%)	873 (41%)	<0.001	2484 (31%)	2916 (39%)	<0.001
Nitrates	6568 (49%)	1150 (54%)	<0.001	3856 (48%)	3861 (51%)	<0.01
CCB	6555 (49%)	1160 (54%)	<0.001	3904 (49%)	3810 (51%)	<0.01
Diuretics	1926 (14%)	449 (21%)	<0.001	1018 (13%)	1356 (18%)	<0.001
Anti-platelets agents	7934 (59%)	1136 (53%)	<0.001	4867 (61%)	4203 (56%)	<0.001
Components of the metabolic syndrome						
FPG >100 mg/dL	5805 (44%)	1412 (67%)	<0.001	1873 (23%)	5347 (72%)	<0.001
Low HDL^b^	9155 (73%)	1858 (90%)	<0.001	4065 (56%)	6948 (94%)	<0.001
TG >150 mg/dL	5226 (40%)	1297 (61%)	<0.001	1198 (15%)	5325 (72%)	<0.001
BMI >30 kg/m^2^	340 (2.6%)	2122 (100%)	<0.001	340 (4%)	2122 (28%)	<0.001
Elevated BP^c^	8978 (68%)	1761 (83%)	<0.001	4329 (54%)	6410 (86%)	<0.001

*BMI* body mass index; *BP* blood pressure; *CHO* total cholesterol; *COPD* chronic obstructive pulmonary disease; *Creat* creatinine; *CVA* cerebral vascular accident; *DM* diabetes mellitus; *FPG* fasting plasma glucose; *HDL* high-density lipoprotein; *HTN* hypertension; *IDF* International Diabetes Federation; *LDL* low density lipoprotein; *MI* myocardial infarction; *NCEP* National Cholesterol Educational Program; *NYHA* New York Heart Association; *TG* triglycerides

^a^Continuous variables are reported as mean ± standard deviation if normally distributed; otherwise, as median with 25th–75th range. Categorical variables are reported as numbers (%)

^b^Low HDL defined as HDL <40 mg/dL in males and HDL <50 mg/dL in females

^c^Systolic blood-pressure >130 mmHg or/and diastolic blood-pressure >85 mmHg

As expected, patients with MetS had an adverse clinical and biochemical profile, including higher incidence of diabetes, hypertension, hypercholesterolemia, and NYHA class >2. According to the IDF definition, patients with the MetS were slightly younger, with a male predominance, and had slightly lower serum creatinine concentration compared to patients without MetS (Table 1).

Prevalence of past CVA, and chronic obstructive lung disease (COPD) were similar. Low-density lipoprotein (LDL) levels and history of past MI were similar when MetS was defined by IDF criteria. When MetS was defined according to the NCEP criteria, past MI rates was similar between groups, however, LDL levels were higher in the MetS group vs. those without MetS (156 ± 36 vs. 154 ± 33, p < 0.001).

Patients with the MetS were significantly more likely to receive beta-blockers, diuretics, calcium channel blockers and nitrates, and less likely to receive antiplatelet therapy (Table 1).

We further compared the individual components of MetS defined by the NCEP vs. the IDF criteria (Additional file 1: Table S1). Patients categorized according to the NCEP definition were more likely to have other metabolic components (hypertension, IFG, hypertriglyceridemia and low HDL) with the exception of BMI >30 that was present in only 28% of the NCEP group vs. 100% of the IDF group (all p value < 0.001).

### Long-term mortality by the presence of MetS

**Fig. 1:**
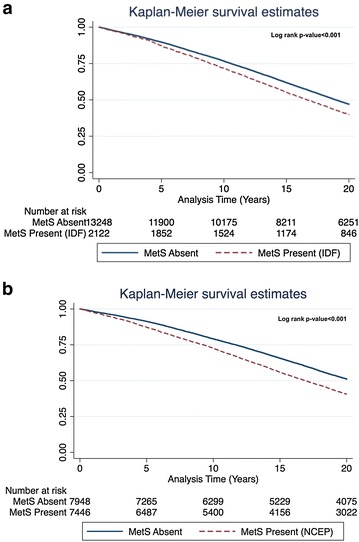
Kaplan-Meier 20-year survival estimates for the entire cohort. **a** Survival estimates according to the presence or absence of the metabolic syndrome according to the IDF definition. **b** Survival estimates according to the presence or absence of metabolic syndrome according to the NCEP definition. *Cre* creatinine; *DM* diabetes mellitus; *IDF* International Diabetes Federation; *LDL* low density lipoprotein; *MetS* metabolic syndrome; *MI* myocardial infarction; *NCEP* National Cholesterol Educational Program

Kaplan–Meier survival analysis showed that at 20 years of follow-up all-cause mortality probability was significantly higher among patients with MetS vs. those without MetS (Fig. 1). When defined by the IDF criteria the respective cumulative mortality probability at 20 years were 1429 (67%) and 8095 (61%) (p < 0.001 for the overall comparison during follow-up; Fig. 1a), and when defined by the NCEP criteria the respective rates were 4987 (67%) and 4329 (54%) (p < 0.001 for the overall comparison during follow-up; Fig. 1b). Notably, separation in event rates between MetS and non-MetS patients appeared after approximately one year and was sustained thereafter.

Consistently, multivariate adjusted for: age, gender, smoking status and major comorbidities (Creatinine >1.5 mg/dL, DM, HTN, past MI, previous CVA and NYHA >2), MetS defined by the NCEP was associated with a significant 21% independent increased mortality risk (HR 1.21; 95% CI 1.13–1.29; Table 2a) whereas MetS defined by the IDF, was similarly independently associated with a 17% increased all-cause mortality risk (HR 1.17; 95% CI 1.07–1.28; Table 2b). Consistent results were obtained when patients randomized to the interventional BIP trial (n = 3090) were excluded or when medications were adjusted for. The mortality risk associated with MetS was similar when BMI was excluded, and patients were defined based only on two out of remaining four criteria.

**Table 2:** Independent all-cause mortality risk predictors in patients with stable CAD using the (a) NCEP and (b) IDF MetS definition

	Adjusted HR	95% CI for upper	*P* value
(a) Hazard ration among the NCEP metabolic group			
Metabolic NCEP	1.21	1.14–1.29	<0.001
Age >65 years	1.08	1.07–1.08	<0.001
Male gender	1.19	1.09–1.31	<0.001
Creatinine >1.5 mg/dL	1.59	1.38–1.84	<0.001
Diabetes mellitus	1.61	1.49–1.74	<0.001
Hypertension	1.08	1.01–1.15	0.01
Past MI	1.41	1.31–1.52	<0.001
Previous CVA	1.07	0.83–1.39	0.59
NYHA >2	1.25	1.10–1.43	<0.001
Active smoker	1.57	1.43–1.71	<0.001
(b) Hazard ration among the IDF metabolic group			
Metabolic IDF	1.17	1.07–1.28	<0.001
Age >65 years	1.08	1.08–1.09	<0.001
Male gender	1.17	1.06–1.29	<0.001
Creatinine >1.5 mg/dL	1.60	1.38–1.84	<0.001
Diabetes mellitus	1.68	1.56–1.81	<0.001
Hypertension	1.10	1.04–1.18	0.003
Past MI	1.41	1.31–1.52	<0.001
Previous CVA	1.10	0.85–1.42	0.48
NYHA >2	1.23	1.07–1.41	0.003
Active smoker	1.57	1.43–1.72	<0.001

Both models further adjusted for: hypertension, smoking status, and severe heart failure (NYHA >2)

*CI* 95% confidence interval; *CVA* cerebral-vascular accident; *HR* hazard ratio; *MI* myocardial infarction; *NCEP* National Cholesterol Education Program; *IDF* International Diabetes Federation; *NYHA* New York Heart Association

Increased serum creatinine, the presence of diabetes mellitus, current smoking and a history of myocardial infarction were additional independent predictors of all-cause mortality (Table 2a, b).

### Subgroup analysis

**Fig. 2:**
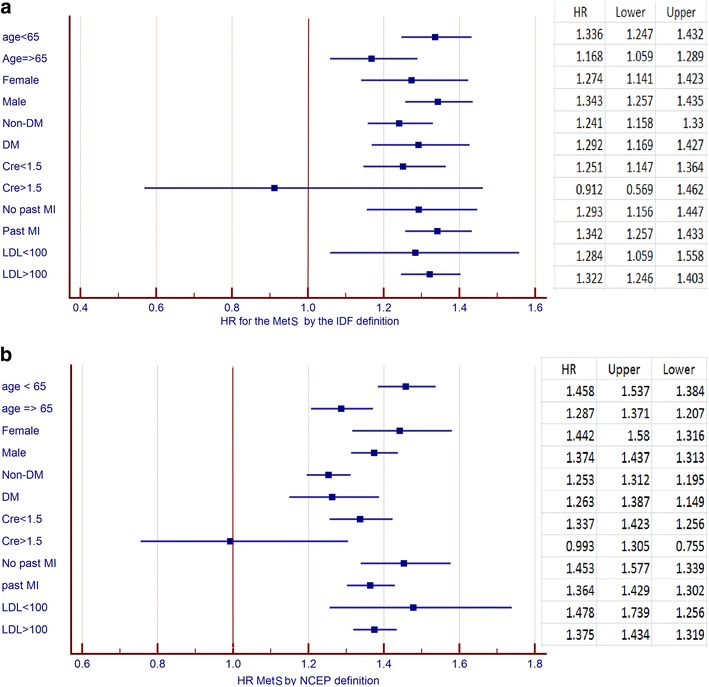
Mortality risk associated with metabolic syndrome presence according to the IDF (**a**) and NCEP definitions (**b**) in pre-specified subgroups. Both models further adjusted for: hypertension, smoking status, and severe heart failure (NYHA >2). *Cre* creatinine; *DM* diabetes mellitus; *IDF* International Diabetes Federation; *LDL* low density lipoprotein; *MetS* metabolic syndrome; *MI* myocardial infarction; *NCEP* National Cholesterol Educational Program

We further explored the independent association between the presence of MetS and long-term mortality in predefined subgroups of patients (Fig. 2). This analysis showed that the mortality risk was increased by 15–25% across all major groups when both definitions of MetS were used, with the exception of patients with creatinine concentration >1.5 mg/dL (p value of interaction p = 0.04) and those 65 years old or older, when NCEP definition was used (p value for interaction = 0.003; Fig. 2a, b).

## Discussion

The primary findings of our study are: (1) MetS is associated with approximately 20% greater all-cause mortality risk at 20-year of follow-up; the risk is independent of other important predictors of adverse outcomes; (2) The two leading definitions of MetS, respectively IDF and NCEP criteria, have similar long-term prognostic implications despite the inclusion of a much greater number of patients according to the NCEP definition; (3) the mortality risk associated with MetS is consistent in most patient subgroups, with the possible exception of those with renal dysfunction and less pronounced in patients aged 65 years or older.

A number of prior large-scale studies demonstrated the long-term prognostic significance of every single metabolic component of the MetS [15, 30–33], whereas controversy remains regarding additional value of the MetS in the risk estimation on top of its individual components [28, 34–39].

Although earlier studies tend to show a significant association between MetS and all-cause mortality especially among middle-aged individuals similar to our cohort, [10, 40–42] they were shorter in duration [17, 20, 43, 44], had smaller samples [45–47], had cardiovascular mortality as their primary outcome and comprised mostly of patients after an intervention [17, 20, 43–47]. In studies by Marso et al. and Miller et al. patients with acute coronary syndrome were enrolled, rather than patients with a stable coronary disease [32, 33].

On the other hand, more recent studies, who enrolled patients with ACS were unable to demonstrate such an association in coronary patients after ACS or revascularization. [48, 49].

The largest meta-analysis that included near one million patients (total n = 951,083) concluded that the MetS is associated with a twofold increase in cardiovascular outcomes and a 1.5-fold increase in all-cause mortality rates [42]. Nevertheless, most subjects included in this analysis had no overt cardiovascular disease.

One recent large study by van Herpt et al. has shown that MetS increased the all-cause mortality in univariate analysis, yet was unable to find any significant associations of MetS with all-cause mortality after adjustment for age, gender and comorbidities. [39].

Additionally, previous studies were limited by their small size and relatively modest follow-up period, which was mostly less than 3 years in the majority of studies concerning the MetS and mortality. [16–21] Furthermore these studies have explored the cardiovascular mortality rather than all-cause mortality as their primary outcome. [16–21].

Hence the enrollment for this study took place between the years 1990 and 1992, the majority of the patients didn’t undergo revascularization at the time of their enrollment. While most studies that evaluated patients after a recent acute coronary syndrome [14, 15, 30] or after coronary revascularization [16–21] failed to show a significant effects of MetS on mortality among patients with stable coronary artery disease and without revascularization, our study did find a significant association with all-cause mortality.

To the best of our knowledge, our study is the largest and presents the longest follow-up period of patients with stable CAD demonstrating an independent association of MetS with all-cause mortality.

Despite the significant numeric difference, and the significant difference of their metabolic components, both MetS groups (IDF and NCEP definitions), had almost a similar effect on the 20-year all-cause mortality outcome, regardless of the definition employed.

Notably, the number of patients with MetS according to NCEP criteria was significantly larger than the number obtained when the IDF criteria were utilized (7446 vs. 2122). This difference is due to the obligatory inclusion of the central obesity as a required according to the IDF criteria, in addition to the two or more of the remaining 4 criteria detailed above. In contrast, the NCEP does not present such an obligatory requirement and is based on the presence of any three criteria. This leads to the fact that all IDF patients are also included in the NCEP group.

Despite the fact that the presence of the MetS possesses a definite predictive value, the view of this metabolic cluster as a prognostic tool only will be too simplistic. MetS is a widely accepted concept regarding a biological condition based on the complex and interrelated pathophysiological mechanisms starting from excess central adiposity and insulin resistance. MetS identifies additional important residual vascular risk mainly associated with insulin resistance, atherogenic dyslipidemia, non-alcoholic fatty liver and type 2 diabetes development. Therefore, the MetS could be a useful additional contributor in estimation of global cardiovascular risk beyond its components and other standard risk factors like age, high LDL-C, etc. [50–55].

Moreover, the concept that the metabolic syndrome is a consequence of obesity and insulin resistance, provides a useful “life-style changes” approach for prevention and treatment: caloric restriction, weight-loss and increased physical activity within cardiac rehabilitation programs for patients with CAD.

## Limitations

Our study has a number of limitations. First, it is a retrospective study that enrolled patients during a period where different treatments were used for controlling blood glucose, hyperlipidemia and hypertension, thus our results warrant validation in more contemporary populations. Second, not all confounders can be accounted for nor were all possible variables measured at enrolment. Third, we have no data regarding clinical events and clinical management after the screening period. Finally, our data lacks waist circumference assessment which is important element of the definition of central obesity as a component of the MetS. However, we replaced this criteria with the BMI >30 according to the consensus of the IDF and NCEP. Furthermore, when we excluded the BMI criteria, and set the diagnosis of MetS as the presence of two out the four remaining criteria, similar results were obtained.

## Conclusions

Metabolic syndrome is independently associated with increased 20-year mortality in patients with stable coronary artery disease. The excess of the very long-term mortality risk was consistent regardless of the MetS definition employed and similar across most population subgroups, yet less pronounced in patients of 65 year or older and absent in patients with renal failure.

## Additional file

**Additional file 1: Table S1.** Differences between the MetS definitions in terms of their individual components.

## Abbreviations

BIP: Bezafibrate Infarction Prevention
BMI: body mass index
CABG: coronary artery bypass grafting
CAD: coronary artery disease
COPD: chronic obstructive pulmonary disease
CVA: cerebral vascular accident
DM: diabetes mellitus
FPG: fasting plasma glucose
HDL: high-density lipoprotein
HTN: hypertension
IDF: International Diabetes Federation
LDL: low density lipoprotein
LML: log minus log
MetS: metabolic syndrome
MI: myocardial infarction
NCEP: National Cholesterol Educational Program
NYHA: New York Heart Association
PTCA: percutaneous transluminal coronary angioplasty
SD: standard deviation
TG: triglycerides
