# Supplementary material for: Metabolic syndrome is independently associated with increased 20-year mortality in patients with stable coronary artery disease
Source: Cardiovasc Diabetol. 2016 Oct 28;15:149. doi: 10.1186/s12933-016-0466-6 (PMC5084328; doi:10.1186/s12933-016-0466-6)
Supplement: Supplementary file 1 — Additional file 1: Table S1. Differences between the MetS definitions in terms of their individual components. [file 12933_2016_466_MOESM1_ESM.docx]

**Additional file 1: Table S1.** Differences between the MetS definitions in terms of their individual components

| Components of the metabolic syndrome | IDF metabolic patients (n = 2122) | NCEP metabolic patients (n = 7446) | *P* value |
| --- | --- | --- | --- |
| FPG >100 mg/dL | 1412 (67%) | 5347 (72%) | <0.001 |
| Low HDL^a^ | 1858 (90%) | 6948 (94%) | <0.001 |
| TG >150 mg/dL | 1297 (61%) | 5325 (72%) | <0.001 |
| BMI >30 kg/m^2^ | 2122 (100%) | 2122 (28%) | <0.001 |
| Elevated blood pressure^b^ | 1761 (83%) | 6410 (86%) | <0.001 |

*BMI* body mass index; *HDL* high-density lipoprotein; *FPG* fasting plasma glucose; *TG* triglycerides

^a^Low HDL defined as HDL <40 mg/dL in males and HDL <50 mg/dL in females

^b^Defined as systolic blood-pressure >130 mmHg or/and diastolic blood-pressure >85 mmHg
